# Functional and oral health–related quality-of-life outcomes 12 months after disc repositioning in women with refractory bilateral disc displacement without reduction

**DOI:** 10.1007/s10006-026-01569-x

**Published:** 2026-05-09

**Authors:** Roberto Ferreira Zanin, Cláiton Heitz, Bruna Morgan Dellagostin, Alexandre Weber, Guilherme Omizzolo, Ricardo Augusto Conci, João Batista Blessmann Weber

**Affiliations:** 1 Oral and Maxillofacial Surgeon, Department of Oral and Maxillofacial Surgery, Pontifical Catholic University of Rio Grande do Sul (PUCRS), 6681 Ipiranga Avenue- Partenon, Porto Alegre, RS Brazil; 2 Department of Oral and Maxillofacial Surgery, Pontifical Catholic University of Rio Grande do Sul (PUCRS), Porto Alegre, RS Brazil; 3 Hospital Universitário Santa Terezinha, Joaçaba, SC Brazil; 4 Department of Oral and Maxillofacial Surgery, State University of Western Paraná (UNIOESTE), Cascavel, PR Brazil

**Keywords:** Oral health, Patient-reported outcomes, Pain, Temporomandibular joint disorders, Disc displacement without reduction, Oral health–related quality of life

## Abstract

**Purpose:**

To evaluate longitudinal changes in oral health–related quality of life (OHRQoL), pain, and mandibular function over 12 months after temporomandibular joint (TMJ) disc repositioning with mini-anchor fixation in women with refractory bilateral disc displacement without reduction (DDwoR).

**Methods:**

In this prospective cohort study, 34 women with bilateral DDwoR confirmed by Diagnostic Criteria for Temporomandibular Disorders (DC/TMD) and magnetic resonance imaging (MRI), refractory to at least 6 months of conservative therapy, were assessed at baseline (T0), 1 month (T1), and 12 months (T2). Outcomes included the Oral Health Impact Profile-14 (OHIP-14), visual analog scale for pain (VAS-P), Helkimo Clinical Dysfunction Index (DI), and mandibular mobility measurements. Longitudinal changes were analyzed using repeated-measures ANOVA and linear mixed-effects models.

**Results:**

Pain (VAS-P) decreased from 7.6 to 0.7, OHIP-14 from 29.4 to 7.0, and DI from 4.1 to 1.4 by 12 months (all *p* < 0.001). Reductions in OHIP-14 and VAS-P exceeded the predefined MCIDs (OHIP-14, ≥ 4.0 points; VAS-P, ≥ 2.0 points on the 0–10 scale). Mandibular mobility also improved significantly over time. Age-group analyses were exploratory; although an overall time × age interaction was observed for OHIP-14, no significant between-group differences were found at postoperative time points.

**Conclusion:**

TMJ disc repositioning with mini-anchor fixation was associated with significant improvements in OHRQoL, pain, and function in women with refractory bilateral DDwoR over 12 months.

**Clinical trial number:**

Not applicable.

## Introduction

Anterior disc displacement is one of the most common temporomandibular disorders (TMDs) with a higher prevalence in women [1]. Women reportedly experience a greater incidence and severity of temporomandibular joint (TMJ) and masticatory muscle dysfunction. The clinical presentations often include clicking, joint pain, limited mouth opening, and functional limitations in chewing, swallowing, and speaking [2–5]. TMDs are also associated with higher levels of depression, anxiety, stress, and reduced oral health-related quality of life (OHRQoL) [6–8].

First-line management of anterior disc displacement without reduction (DDwoR) is conservative − stabilization splints, physiotherapy, and pharmacologic therapy [5] − and remains standard dental practice. However, a subset of patients does not improve after a well-conducted course of conservative therapy and continues to report pain, dysfunction, and restricted mandibular mobility [4].

For those patients, TMJ disc repositioning with mini-anchor fixation may offer an alternative for improving both function and OHRQoL [9–12]. As OHRQoL reflects perceived physical and mental health within a sociocultural context [13], accurate evaluation is important. Patient-reported outcomes such as Oral Health Impact Profile-14 (OHIP-14), Helkimo clinical dysfunction index (DI), and Visual Analog Scale for Pain (VAS-P) provide valuable information, and the addition of these instruments may enhance clinical assessment and longitudinal follow-up [14–18]. However, subjective tools such as the OHIP-14 and VAS-P are susceptible to interpretative and recall bias [19].

Standardized TMD assessment is essential for evaluating severity, comparing populations, and monitoring treatment outcomes [20]. Yet the interplay between subjective (OHRQoL and pain) and objective (clinical dysfunction and mandibular mobility) indicators, and their influence on OHRQoL, remains underexplored. Although prior studies have assessed OHRQoL in TMD using various instruments [4, 6, 14–16], to our knowledge, none has integrated the OHIP-14, DI, and VAS-P to evaluate bilateral DDwoR in women undergoing disc repositioning with mini-anchors. Surgical disc repositioning may be considered for carefully selected patients with persistent symptoms after failure of conservative therapy. Although the relationship between disc position and symptoms remains debated [21], surgical intervention has been associated with improvements in pain, mandibular function, and patient-reported outcomes in selected cases [9–12, 22].

This study aimed to assess the impact of DDwoR on OHRQoL and to determine whether TMJ disc repositioning with mini-anchor improves OHIP-14, DI, and VAS-P scores. We hypothesized these scores would improve after intervention.

## Materials and methods

### Ethical aspects

This single-center study was conducted at the Department of Oral and Maxillofacial Surgery of Pontifical Catholic University of Rio Grande do Sul (PUCRS), Brazil, and was approved by the Institutional Research and Ethics Committee (CAAE 92776018.3.0000.5336). It was conducted in accordance with the Declaration of Helsinki and adhered to the Strengthening the Reporting of Observational Studies in Epidemiology (STROBE) guidelines for reporting observational studies. Informed consent and assent were obtained from all participants and, where applicable, from their legal guardians. This prospective cohort included patients with documented failure of prolonged conservative therapy. The absence of a concurrent non-surgical control group limits causal inference, and the findings should therefore be interpreted as longitudinal clinical outcomes observed after the intervention.

### Participants

Eligible participants were women aged ≥ 16 diagnosed with bilateral TMJ DDwoR refractory to at least 6 months of conservative therapy, who were assessed for surgical treatment between July 2021 and January 2024. Those who met all eligibility criteria and underwent disc repositioning with mini-anchor fixation were included in the prospective cohort. The inclusion of patients ≥ 16 years reflects the clinical reality of symptomatic DDwoR management, as most craniofacial growth is near completion in late adolescence.

Patients with condylar ankylosis, TMD-related systemic diseases (rheumatoid arthritis, myasthenia gravis, Ehlers-Danlos syndrome), or previous TMJ surgery were excluded. Participants were clinically diagnosed according to the Diagnostic Criteria for Temporomandibular Disorders (DC/TMD) Axis I. The diagnosis of DDwoR, with or without limited opening, was confirmed in all cases by magnetic resonance imaging (MRI).

Data were collected at baseline (T0), 1 month (T1), and 12 months (T2) after surgery. Clinical findings were consistent with chronic mechanical overload and progressive biomechanical dysfunction associated with long-standing DDwoR, as evidenced by limited translational movement, persistent closed lock when present, and increased loading on the posterior attachment on clinical and MRI examination.

### Conservative management before surgical indication

Before surgical indication, all patients underwent a standardized conservative management protocol for a minimum of six months. This multidisciplinary approach included stabilization splint therapy using a hard acrylic (Michigan-type splint) worn primarily during nighttime, patient education and self-management strategies (including avoidance of parafunctional habits and adoption of a soft-food diet), short courses of anti-inflammatory and/or muscle-relaxant medications when clinically indicated, and home-based guided jaw exercises aimed at maintaining mandibular mobility. Surgical intervention was considered only for patients who remained refractory to these measures, characterized by persistent pain (VAS-*P* > 4), clinical dysfunction (Helkimo DI > 3), and a documented negative impact on oral health–related quality of life (OHIP-14) during the conservative treatment period.

### Sample

The sample size was conservatively estimated using a two-sided α of 0.05 and 90% power. For the OHIP-14 outcome, a baseline (T0) standard deviation of 11.6, a 1-month (T1) standard deviation of 6.0, and an expected T0–T1 mean difference of 9.2, re-expressed on the conventional 0–56 scale from previous data [9], were used for the sample size calculation. The estimated minimum sample size was 24 patients (WinPepi, v.11.65; PEPI-for-Windows, 2016).

### Clinical assessment

Data were collected preoperatively (T0), at 1 month (T1), and at 12 months (T2) after TMJ disc repositioning with mini-anchor fixation. Thirty-four women aged 16–71 years were included (mean age 40 years, SD 15.5) and categorized into two age groups: ≤40 years (*n* = 19) and > 40 years (*n* = 15). The patient (bilateral DDwoR) was the unit of analysis.

### Follow-up and retention

All 34 participants attended both follow-ups at T1 and T2. Retention was optimized by pre-scheduled appointments before discharge, reminder calls/text messages, flexible rescheduling within a predefined visit window, and continuity of care with the same surgeon and team.

### Outcomes

To reduce selection and measurement bias, we used prospective assessment at fixed time points, validated instruments (OHIP-14, DI, VAS-P), standardized measurement protocols, and the same surgeon/technique for all cases.

#### The oral health impact profile (OHIP-14)

Participants completed the validated Portuguese version of the OHIP-14 [23], which includes 14 items covering seven OHRQoL domains: functional limitation, physical pain, psychological discomfort, physical disability, psychological disability, social disability, and handicap. Each item is scored on a 0–4 Likert scale (0 = never; 1 = hardly ever; 2 = occasionally; 3 = fairly often; 4 = very often). Domain scores were calculated by summing the two items within each domain, yielding domain scores ranging from 0 to 8. The overall OHIP-14 score was obtained by summing all 14 item scores, yielding a total score ranging from 0 to 56. Higher scores indicate greater negative impact on OHRQoL [16].

The predefined minimal clinically important difference (MCID) for within-subject improvement was a reduction of at least 4 points.

#### Helkimo clinical DI (0–5)

The Helkimo Clinical Dysfunction Index (DI) was used to assess functional impairment [17]. Five items were evaluated: (a) limited mandibular mobility; (b) joint function with deviations, joint sounds, or locking; (c) TMJ pain during jaw movement; (d) muscle pain; and (e) pain on palpation. Each item was scored as 0 (no dysfunction), 1 (mild dysfunction), or 5 (severe dysfunction). The total score (range 0–25) was subsequently classified into dysfunction categories according to Helkimo’s criteria: DI 0 (0), DI 1 (1–4), DI 2 (5–9), DI 3 (10–13), DI 4 (15–17), and DI 5 (20–25). For descriptive and longitudinal analyses, the mean DI category score (0–5) reported in the tables and figures represents the average of these categorical levels across participants.

#### Mandibular mobility

Maximum opening, lateral excursions, and protrusion were measured using a digital caliper (accuracy: 0.1 mm).

#### Pain assessment

Patients rated TMJ pain intensity using a 10–point VAS-P [18] at T0, T1, and T2. The MCID was predefined as a reduction of at least 2 points on the 0–10 VAS-P.

In addition to the VAS-P, the Helkimo DI incorporates functional and palpation-based pain assessments. Pain on palpation of the TMJ and masticatory muscles, as well as pain during mandibular movements (opening, laterality, protrusion), was evaluated at T0, T1, and T2 as part of the DI subitems (b–e) as described in Section Helkimo clinical DI (0–5).

### Surgical technique

All procedures were performed by a single surgeon under general anesthesia via a standard endaural approach. After capsulotomy and release of lateral, medial, and anterior attachments, the disc was mobilized and repositioned passively over the condylar head. Fixation was achieved with a self-drilling suture mini-anchor (Ancortec, Materialise Brazil) placed on the posterior condylar surface 8–10 mm below the condylar head and connected to the TMJ disc using a horizontal mattress configuration with a double 2 − 0 non-resorbable polyester suture. Disc position and mandibular range of motion were verified intraoperatively; routine postoperative MRI was not performed.

### Adverse events

Complications and reinterventions were prospectively recorded at 1 week, T1, and T2, including surgical-site infection, hematoma, transient facial nerve paresis, hypoesthesia, device/anchor failure, joint locking, and reoperation.

### Postoperative care

Postoperative care included a 14-day liquid-to-pureed diet, transitioning to soft foods by week 6. Physiotherapy was initiated within the first postoperative week and maintained for at least 6 weeks, consisting of supervised sessions (1–3 sessions per week) and home exercises (5–10 times daily). Stabilization splints were not used routinely and were reserved only for patients with significant bruxism or residual myalgia to protect the surgical site and manage parafunctional forces. No additional temporomandibular joint interventions or pharmacologic treatments were routinely prescribed during the follow-up (T1–T2) period beyond the standard postoperative care described above.

### Statistical analysis

Normality was assessed using the Kolmogorov-Smirnov test. Outcome variables are summarized as mean (SD) with 95% confidence intervals (CIs). The primary analysis used repeated-measures ANOVA with Bonferroni-adjusted pairwise comparisons to test within-subject changes across T0–T2 for OHIP-14, VAS-P, DI, and mandibular mobility. To evaluate differences in outcome trajectories by age (≤ 40 vs. >40), linear mixed-effects models were subsequently fitted, assessing the fixed effects of time, age group, and the time × age interaction. Age-group analyses were conducted as exploratory, hypothesis-generating analyses. The 40-year cut-point was selected for clinical interpretability and to contrast younger and older adult subgroups, given the higher prevalence of disc displacement in women aged 20–40 years and the greater likelihood of degenerative changes in older patients. As a sensitivity analysis for the ordinal Helkimo DI category score (0–5), within-subject changes across T0, T1, and T2 were also assessed using the Friedman test, followed by Bonferroni-adjusted Wilcoxon signed-rank tests for pairwise comparisons. Effect sizes were paired Cohen’s d (OHIP-14) and Glass’s Δ (VAS-P, DI; baseline SD), each with 95% CIs. Adverse-event proportions are reported with Wilson 95% CIs. Analyses were performed in IBM SPSS Statistics v23.0 (IBM Corp., Armonk, NY) with significance set at 5%.

## Results

**Fig. 1:**
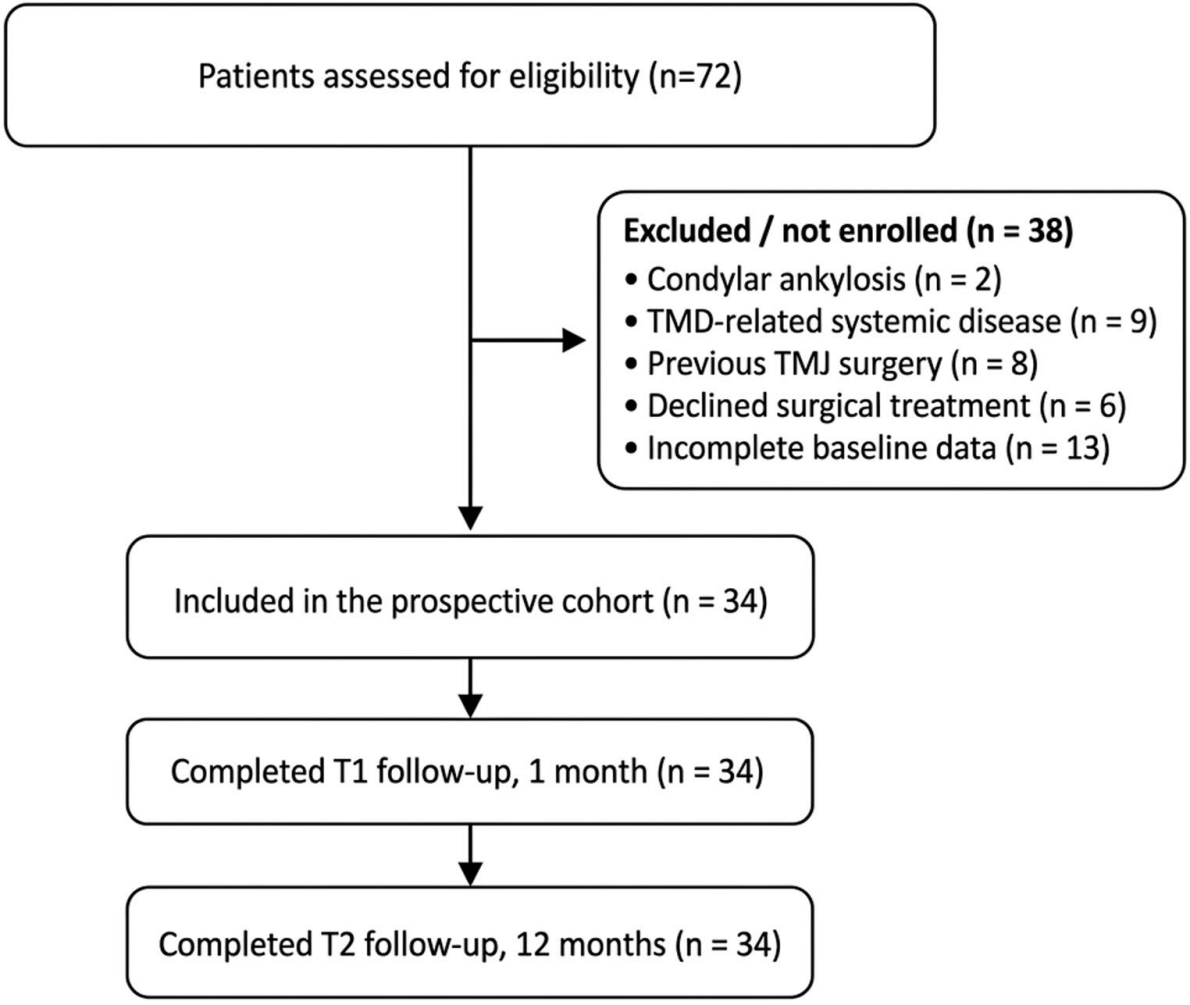
Flow diagram of patient selection and follow-up

A total of 72 patients were assessed for eligibility between July 2021 and January 2024. After exclusion or non-enrollment (Fig. 1), 34 women (age range, 16–71 years; mean [SD], 40.0 [15.5] years) were included in the prospective cohort. All participants completed the 1-month (T1) and 12-month (T2) follow-up assessments, with no missing outcome data (Tables 1, 2, 3, 4 and 5).

**Table 1:** Sample distribution by age group at baseline, *n* (%)

	Age (Years)		
	Group I	Group II	Total
	≤ 40 Years	> 40 Years	
N	19	15	34
%	55.9	44.1	100

**Table 2:** Distribution of OHIP-14 item responses by domain at baseline (T0), 1 month (T1), and 12 months (T2) (%)

Domain	Item	OHIP-14	T0 Before surgery					T1 1 month					T2 12 months				
		Question	0	1	2	3	4	0	1	2	3	4	0	1	2	3	4
Functional limitation	**1**	Have you had trouble pronouncing words because of problems with your mouth or joint?	52.9	2.9	23.5	14.7	5.9	55.9	20.6	23.5	0	0	85.3	14.7	0	0	0
	**2**	Have you felt your sense of taste has worsened because of problems with your mouth or joint?	73.5	8.8	8.8	5.9	2.9	82.3	8.8	8.8	0	0	88.2	5.9	2.9	2.9	0
Physical pain	**3**	Have you had painful aching in your mouth because of problems with your mouth or joint?	0	5.9	5.9	47	41.2	5.9	47	47	2.9	0	44.1	47	8.8	2.9	0
	**4**	Have you found it uncomfortable to eat any foods because of problems with your mouth or joint?	2.9	2.9	11.8	44.1	38.3	2.9	47	32.3	17.6	2.9	50	47	2.9	2.9	0
Psychological discomfort	**5**	Have you been self-conscious because of problems with your mouth or joint?	2.9	2.9	8.8	41.2	44.1	32.3	47	17.6	5.9	0	61.8	29.4	5.9	5.9	0
	**6**	Have you felt tense because of problems with your mouth or joint?	8.8	5.9	11.8	23.5	50	38.3	38.3	17.6	5.9	2.9	58.8	32.3	11.8	0	0
Physical disability	**7**	Has your diet been unsatisfactory because of problems with your mouth or joint?	2.9	2.9	32.3	23.5	38.3	5.9	47	35.3	11.8	2.9	52.9	35.3	14.7	0	0
	**8**	Have you had to interrupt meals because of problems with your mouth or joint?	8.8	2.9	20.6	38.3	29.4	26.4	41.2	29.4	2.9	2.9	61.8	35.3	5.9	0	0
Psychological disability	**9**	Have you found it difficult to relax because of problems with your mouth or joint?	5.9	8.8	23.5	32.3	29.4	14.7	50	32.3	2.9	2.9	47	38.3	14.7	2.9	0
	**10**	Have you been a bit embarrassed because of problems with your mouth or joint?	61.8	2.9	17.6	2.9	14.7	79.4	14.7	5.9	2.9	0	88.2	8.8	2.9	2.9	0
Social disability	**11**	Have you been a bit irritable with other people because of problems with your mouth or joint?	55.9	14.7	11.8	5.9	11.8	64.7	26.4	5.9	2.9	2.9	73.5	20.6	5.9	2.9	0
	**12**	Have you had difficulty doing your usual jobs because of problems with your mouth or joint?	41.2	11.8	26.4	5.9	14.7	38.3	35.3	20.6	5.9	5.9	67.6	23.5	11.8	0	0
Handicap	**13**	Have you felt that life in general was less satisfying because of problems with your mouth or joint?	44.1	11.8	14.7	5.9	23.5	67.6	14.7	17.6	2.9	0	79.4	14.7	8.8	0	0
	**14**	Have you been totally unable to function because of problems with your mouth or joint?	76.4	2.9	11.8	5.9	2.9	70.6	5.9	23.5	2.9	0	85.3	11.8	5.9	0	0

Values are percentages of responses within each item. 0 = never; 1 = hardly ever; 2 = occasionally; 3 = fairly often; 4 = very often. OHIP-14, Oral Health Impact Profile-14

**Table 3:** Distribution of OHIP-14 item responses by domain at baseline (T0), 1 month (T1), and 12 months (T2) (%)

	OHIP-14 SCORES											
	Group I (*N* = 19)				Group II (*N* = 15)				Total (*N* = 34)			
	≤ 40 years				> 40 years							
Domain	T0	T1	T2		T0	T1	T2		T0	T1	T2	
	Score Mean	Score Mean	Score Mean		Score Mean	Score Mean	Score Mean		Score Mean	Score Mean	Score Mean	
	(SD)	(SD)	(SD)	P	(SD)	(SD)	(SD)	P	(SD)	(SD)	(SD)	P
Functional Limitation	1.4 (2.2)^A^	0.8 (1.6)^A^	0.2 (1.0)^B^	0.001	2.2 (2.8)^A^	1.0 (1.6)^AB^	0.6 (1.0)^B^	< 0.001*	1.8 (2.6)^A^	1.0 (1.6)^AB^	0.4 (1.0)^B^	< 0.001*
Physical Pain	6.4 (1.6)^A^	3.2 (1.4)^B^	1.4 (1.4)^C^	< 0.001*	6.4 (2.0)^A^	3.2 (1.8)^B^	1.2 (1.6)^C^	< 0.001*	6.4 (1.8)^A^	3.2 (1.6)^B^	1.4 (1.4)^C^	< 0.001*
Psychological Discomfort	6.2 (2.0)^A^	1.8 (1.4)^B^	1.0 (1.4)^B^	< 0.001*	6.4 (2.6)^A^	2.4 (2.2)^B^	1.2 (1.8)^B^	< 0.001*	6.2 (2.2)^A^	2.0 (1.8)^B^	1.2 (1.6)^B^	< 0.001*
Physical Disability	5.6 (2.0)^A^	2.4 (2.0)^B^	1.0 (1.2)^C^	< 0.001*	5.6 (2.4)^A^	3.2 (1.8)^B^	1.2 (1.6)^C^	< 0.001*	5.6 (2.2)^A^	2.8 (1.8)^B^	1.0 (1.4)^C^	< 0.001*
Psychological Disability	3.8 (3.2)^A^	1.6 (2.0)^B^	0.8 (1.4)^B^	< 0.001*	3.8 (3.2)^A^	1.6 (1.8)^B^	1.2 (1.8)^B^	< 0.001*	3.8 (3.2)^A^	1.6 (1.8)^B^	1.0 (1.6)^B^	< 0.001*
Social Disability	2.8 (3.2)^A^	1.6 (2.4)^AB^	0.6 (1.2)^B^	< 0.001*	2.0 (2.4)^A^	1.8 (2.0)^A^	1.0 (1.8)^B^	0.001*	2.4 (2.8)^A^	1.6 (2.2)^AB^	0.8 (1.4)^B^	< 0.001*
Handicap	2.4 (3.2)^A^	1.0 (1.8)^AB^	0.4 (0.8)^B^	< 0.001*	1.8 (2.6)^A^	1.4 (2.0)^AB^	0.8 (1.6)^B^	0.012*	2.0 (3.0)^A^	1.2 (1.8)^AB^	0.6 (1.2)^B^	< 0.001*
Total OHIP-14	28.6 (11.4)^A^	13.8 (8.2)^B^	6.0 (5.2)^C^	< 0.001*	30.6 (12.6)^A^	17.8 (12.6)^B^	8.4 (9.8)^C^	< 0.001*	29.4 (11.8)^A^	15.6 (10.4)^B^	7.0 (7.6)^C^	< 0.001*

Different superscript letters within the same row indicate significant Bonferroni-adjusted pairwise differences over time within each group (*p* < 0.05). P values refer to the overall effect of time. OHIP-14 domain scores are presented on the conventional 0–8 scale, and the total OHIP-14 score is presented on the conventional 0–56 scale. SD, standard deviation

**Table 4:** Comparison of OHIP-14, DI, and VAS-P at T0, T1, and T2 by age group

Group		Outcomes						
		T0	T1	T2	T0 – T1	T0 – T2	Δ (T2 − T0)	*P*
		Mean	Mean	Mean	Reduction from baseline %	Reduction from baseline %		
		(SD)	(SD)	(SD)				
Total	OHIP-14	29.4 (11.8)^A^	15.6 (10.4)^B^	7.0 (7.6)^C^	47% reduction	76% reduction	−22.4	< 0.001*
	DI	4.1 (0.9)^A^	2.4 (1.0)^B^	1.4 (0.8)^C^	41% reduction	66% reduction	−2.7	< 0.001*
	VAS-P	7.6 (1.6)^A^	2.3 (0.9)^B^	0.7 (0.8)^C^	70% reduction	91% reduction	−6.9	< 0.001*
≤ 40 years	OHIP-14	28.6 (11.4)^A^	13.8 (8.2)^B^	6.0 (5.2)^C^	52% reduction	79% reduction	−22.6	< 0.001*
	DI	4.1 (0.9)^A^	2.5 (1.0)^B^	1.4 (1.0)^C^	39% reduction	66% reduction	−2.7	< 0.001*
	VAS-P	7.1 (1.7)^A^	2.0 (0.8)^B^	0.6 (0.8)^C^	72% reduction	92% reduction	−6.5	< 0.001*
	OHIP-14	30.6 (12.6)^A^	17.8 (12.6)^B^	8.4 (9.8)^C^	42% reduction	73% reduction	−22.2	< 0.001*
> 40 years	DI	4.1 (0.9)^A^	2.4 (0.9)^AB^	1.3 (0.5)^B^	41% reduction	68% reduction	−2.8	< 0.001*
	VAS-P	8.3 (1.3)^A^	2.7 (0.9)^B^	0.7 (0.9)^C^	67% reduction	92% reduction	−7.6	< 0.001*

Different superscript letters within the same outcome indicate significant Bonferroni-adjusted pairwise differences over time (*p* < 0.05). P values refer to the overall effect of time. Reduction from baseline is expressed relative to T0; Δ indicates change from T0 to T2. OHIP-14 total score is presented on the conventional 0–56 scale. OHIP-14, Oral Health Impact Profile-14; DI, Helkimo Clinical Dysfunction Index; VAS-P, Visual Analog Scale for Pain; SD, standard deviation

**Table 5:** Mandibular mobility (mm) over time in Group I, Group II, and the total sample

	Mandibular Mobility											
	Group I (*N* = 19)				Group II (*N* = 15)				Total (*N* = 34)			
	≤ 40 years				> 40 years							
	T0	T1	T2		T0	T1	T2		T0	T1	T2	
	Mean	Mean	Mean		Mean	Mean	Mean		Mean	Mean	Mean	
	(SD)	(SD)	(SD)	P	(SD)	(SD)	(SD)	P	(SD)	(SD)	(SD)	P
Maximum opening	33.2 (6.1)^A^	21.6 (4.2)^B^	35.2 (4.0)^A^	< 0.001*	30.4 (4.7)^A^	21.5 (3.8)^B^	36.1 (2.8)^C^	< 0.001*	31.9 (5.7)^A^	21.6 (3.9)^B^	35.6 (3.5)^C^	< 0.001*
Lateral right excursion	3.7 (2.2)^A^	0.7 (1.2)^B^	4.9 (1.6)^C^	< 0.001*	2.9 (1.9)^A^	0.7 (0.8)^B^	5.1 (1.7)^C^	< 0.001*	3.4 (2.1)^A^	0.7 (1.0)^B^	5.0 (1.6)^C^	< 0.001*
Lateral left excursion	3.9 (2.2)^A^	0.5 (0.8)^B^	4.3 (1.4)^A^	< 0.001*	3.1 (1.9)^A^	0.7 (0.8)^B^	5.2 (1.9)^C^	< 0.001*	3.6 (2.1)^A^	0.6 (0.8)^B^	4.7 (1.7)^C^	< 0.001*
Maximum protrusion	2.0 (1.4)^A^	0.4 (1.0)^B^	3.6 (1.1)^C^	< 0.001*	1.6 (1.5)^A^	0.3 (0.6)^B^	3.5 (0.9)^C^	< 0.001*	1.8 (1.4)^A^	0.4 (0.8)^B^	3.6 (1.0)^C^	< 0.001*

Different superscript letters within the same row and group indicate significant Bonferroni-adjusted pairwise differences over time (*p* < 0.05). P values refer to the overall effect of time. All overall time effects were significant (*p* < 0.001). SD, standard deviation

### OHIP-14

At T0, most patients reported high impact (scores 3–4) in physical pain (88.2%), psychological discomfort (85.3%), physical disability (67.7%), and psychological disability (61.7%) (Table 2). In contrast, minimal impact (scores 0–1) predominated for functional limitation (82.3%), handicap (79.3%), and social disability (70.6%) (Table 2). A shift was observed at T2, where most participants scored 0–1 for physical pain (97.1%), psychological discomfort (91.2%), physical disability (97.1%), and psychological disability (97.0%). Smaller proportions scored 3 for psychological discomfort (5.9%), psychological disability (2.9%), and physical pain (2.9%), while no participant scored 4 in any domain (Table 2).

#### OHIP-14 total (0–56)

**Fig. 2:**
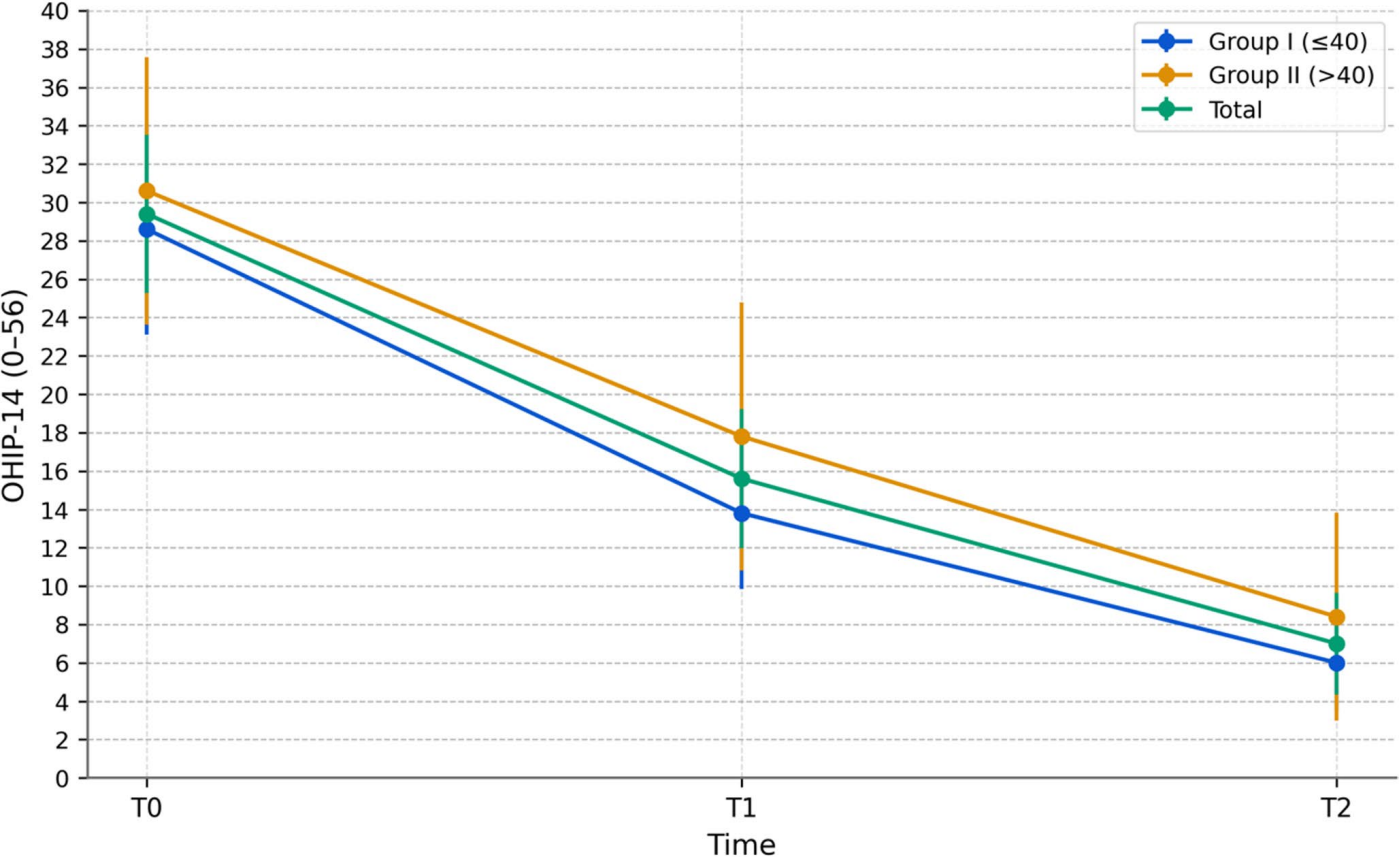
OHIP-14 over time (95% CI). OHIP-14 total score (0–56) at baseline (T0), 1 month (T1), and 12 months (T2) after TMJ disc repositioning with mini-anchor fixation. Lines show Group I (≤ 40 years, *n* = 19), Group II (> 40 years, *n* = 15), and Total (*n* = 34). Points represent means, and error bars denote 95% Cis

**Fig. 3:**
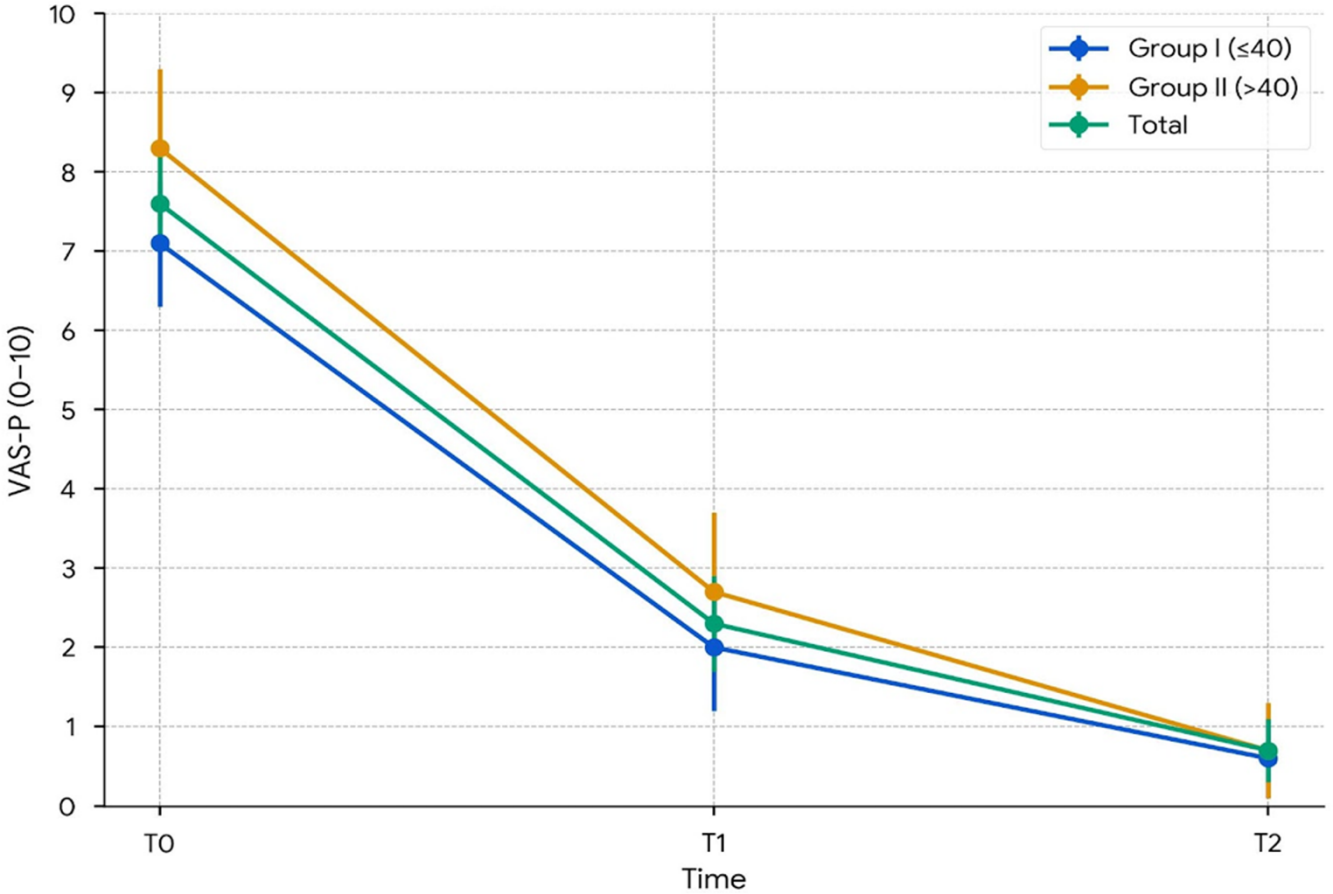
VAS-P over time (95% CI). Pain intensity on a 0–10 points visual analog scale at T0, T1, and T2. Lines show Group I (≤ 40 years, *n* = 19), Group II (> 40 years, *n* = 15), and Total (*n* = 34). Points represent means, and error bars denote 95% CIs

Across groups, physical pain, psychological discomfort, physical disability, and psychological disability all decreased from T0 to T1 (Bonferroni *p* < 0.001; Table 3). Overall, both OHIP-14 and VAS-P scores showed consistent, sustained improvement from T0 to T2 (Bonferroni-adjusted pairwise *p* < 0.001; Figs. 2 and 3; Table 4). The OHIP-14 total mean scores decreased from 29.4 at T0 to 15.6 at T1 and 7.0 at T2 (both Bonferroni-adjusted *p* < 0.001), marking a 76% reduction from baseline (Table 4; Fig. 2). Paired Cohen’s d indicated significant effects: 1.24 (95% CI: 0.79–1.68) for T0–T1 and 2.16 (95% CI: 1.55–2.78) for T0–T2. Both time points exceeded the prespecified MCID (≥ 4.0 points).

### Pain (VAS-P, 0–10 points)

Pain intensity (VAS-P) decreased from 7.6 (SD 1.6; 95% CI: 7.06–8.14) at T0 to 2.3 (SD 0.9; 95% CI: 2.00–2.60) at T1 and 0.7 (SD 0.8; 95% CI: 0.43–0.97) at T2 (all Bonferroni *p* < 0.001), corresponding to 70% (T1) and 91% (T2) reductions (Fig. 3; Table 4). Standardized changes (Glass’s Δ, baseline SD = 1.60) were − 3.31 (T0–T1) and − 4.31 (T0–T2), indicating substantial effects. The mean decreases also surpassed the MCID of ≥ 2 points on the 0–10 VAS-P, confirming the clinical significance of these findings.

### Helkimo clinical DI (0–5)

**Fig. 4:**
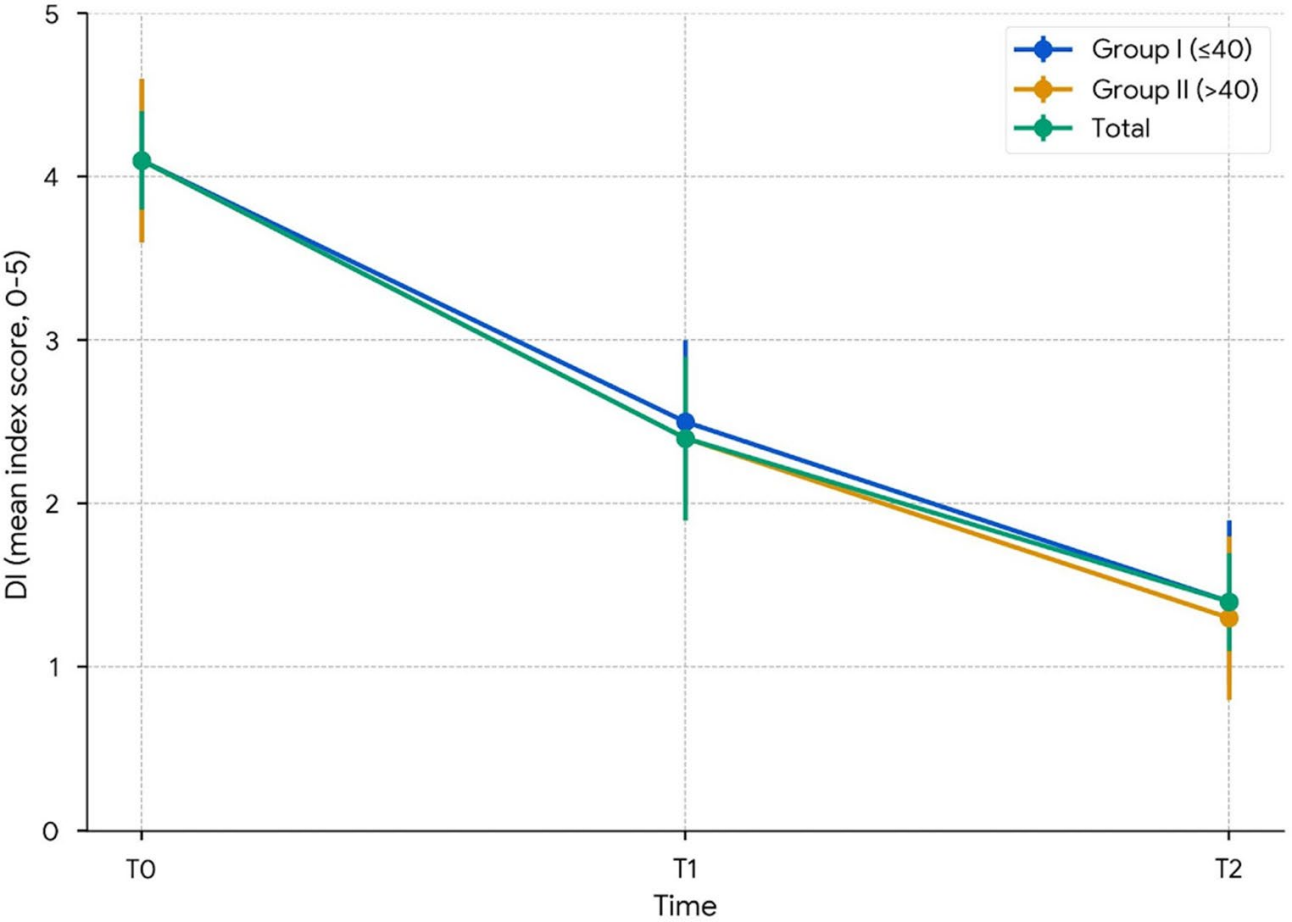
Helkimo clinical dysfunction index over time (95% CI). DI scores at T0, T1, and T2. Lines show Group I (≤ 40 years, *n* = 19), Group II (> 40 years, *n* = 15), and Total (*n* = 34). The y-axis is displayed from 0 to 5 to reflect the observed range. Points represent means, and error bars denote 95% CIs

Mean (SD) scores declined from 4.1 (0.9) at T0 to 2.4 (1.0) at T1, and to 1.4 (0.8) at T2. The 95% CIs were 3.82–4.41, 2.18–2.88, and 1.11–1.66 (Table 4; Fig. 4). Using Glass’s Δ (baseline SD = 0.88), effect sizes were − 1.89 for T0–T1 and − 3.00 for T0–T2. Within groups, all contrasts (T0–T1, T0–T2, T1–T2) were significant in group I. In group II, only T0–T2 remained significant after Bonferroni adjustment, while T0–T1 did not reach significance. A non-parametric sensitivity analysis confirmed the same longitudinal pattern for the ordinal DI category score. The Friedman test showed a significant overall change across T0, T1, and T2 (χ²(2) = 50.51, *p* < 0.001), and Bonferroni-adjusted Wilcoxon signed-rank tests showed significant differences for all pairwise comparisons (T0 vs. T1, *p* < 0.001; T0 vs. T2, *p* < 0.001; T1 vs. T2, *p* < 0.001).

### Mandibular mobility

Assessment showed increased mandibular mobility from T0 to T2 in all measures. Maximum mouth opening from 31.9 to 35.6 mm. Right and left laterality increased from 3.4 to 3.6 mm to 5.0 and 4.7 mm, respectively, while protrusion increased from 1.8 to 3.6 mm. All changes were statistically significant (*p* < 0.001; Table 5).

### Exploratory age-group mixed-model analysis

In exploratory mixed-model analyses, the effect of time was significant for all three outcomes (*p* < 0.001). For OHIP-14, both the overall age effect (*p* = 0.042) and the time × age interaction (*p* = 0.017) were significant; however, no significant between-group differences were observed at the postoperative time points (T1 and T2). For DI, neither the age effect (*p* = 0.724) nor the time × age interaction (*p* = 0.817) was significant. For VAS-P, participants > 40 years reported higher baseline pain scores (*p* = 0.028), but no significant between-group differences were found at T1 or T2, with both groups converging to minimal pain levels by T2 (≤ 40: 0.6; >40: 0.7; Table 4). Given the absence of significant postoperative pairwise contrasts, the OHIP-14 interaction should be interpreted cautiously as exploratory and hypothesis-generating rather than as evidence of a clinically meaningful age-related difference in postoperative outcomes.

### Adverse events

Transient temporal-branch hypoesthesia occurred in 4/34 patients (11.8%; 95% CI 4.7–26.6), resolving within 30 days. Expected postoperative symptoms-moderate pain, edema, and temporary difficulty eating-were limited to the first postoperative week. No infections, hematomas, device/anchor failures, joint locking, or reoperations were observed.

## Discussion

Persistent TMJ symptoms may remain despite well-conducted conservative treatment. In these cases, a transition from a purely chronic-pain disease approach to a musculoskeletal disorder and imaging-guided evaluation is indicated [2]. This process can identify patients with intra-articular disease, such as imaging-confirmed DDwoR with a preserved disc, which may be considered for surgical intervention after failure of conservative treatment.

In this study, we report 12-month associations between disc repositioning and improvements in OHRQoL, pain, and mandibular function in an appropriately triaged cohort of women refractory to conservative treatment. Due to the multifactorial nature of TMD and the higher frequency of bilateral anterior DDwoR in women, only female participants were included to minimize confounding variables. One of the study’s strengths was the retention of all 34 patients over the 12-month period, which was achieved by implementing reminders, offering flexible scheduling, and maintaining close patient engagement.

This full compliance enhances the reliability of the results by reducing potential biases associated with loss to follow-up. The profile of our patients was consistent with what epidemiological studies have already described [3, 18], which have demonstrated that disc displacement is most prevalent among women aged 20–40 years.

In contrast, osteoarthritis (OA) is more common and severe in older adults due to degenerative changes and reduced treatment responsiveness [24]. These factors justified the inclusion of both groups in our analysis. Although participants > 40 years reported higher baseline pain, age-group analyses were exploratory; for OHIP-14, an overall time × age interaction was observed, but no significant between-group differences were found at postoperative time points, and these findings should therefore be interpreted cautiously as hypothesis-generating.

Moreover, baseline OHIP-14 scores indicated substantial physical pain and psychological discomfort, consistent with previous findings [14]. Although patients reported significant eating-related issues, baseline scores for functional limitation, handicap, and social disability were relatively low, indicating a moderate initial impairment in OHRQoL (Table 2). At the 1–month follow-up (T1), improvements in clinical status (DI) were accompanied by subjective improvements in OHRQoL, as measured by the OHIP-14. However, it is important to note that some residual pain and discomfort were still reported (Table 3), suggesting that inflammatory processes, tissue readaptation, and postoperative pain may persist for up to 30 days after surgery.

Consistent with WHO-defined OHRQoL principles [13], improvements in psychological and social domains were observed following functional recovery. Prior studies have linked higher OHIP-14 and DI scores to poorer OHRQoL in TMD populations [25, 26].

In this study, DI, VAS-P, and OHIP-14 scores significantly improved over time (*p* < 0.001), supporting an association between functional and psychosocial outcomes. The progressive and statistically significant decrease in OHIP-14 scores from baseline to 12 months (*p* < 0.001) supports the clinical relevance of the procedure from the patient’s perspective, consistent with another report that found a significant decrease in OHIP-14 scores 3–6 months after the same procedure [9].

However, despite these overall significant improvements at the final follow-up (T2), a few patients still reported minor residual symptoms across several domains. These residual symptoms may result from delayed surgical intervention following the onset of symptoms. Previous studies have shown that TMJ disc repositioning has a 90% success rate within the first 4 years, decreasing to approximately 68% thereafter, suggesting that symptom duration and the structural condition of the joint at the time of surgery influence treatment predictability [27].

In symptomatic DDwoR, prolonged displacement may change joint biomechanics and increase mechanical loading on the posterior attachment and condylar surface. Recent randomized clinical evidence demonstrates that disc position directly affects condylar bone modeling and functional joint loading patterns [28], indicating that structural adaptations become progressively more pronounced with longer symptom duration.

Therefore, for patients who remain symptomatic despite structured conservative therapy, surgical intervention may represent a treatment option in carefully selected refractory cases [27].

Nevertheless, the literature is currently inconclusive regarding which treatment modality offers more effective and long-term gains for DDwoR [29].

Comparing the present findings with outcomes reported for less invasive approaches in DDwoR suggests substantial improvements in pain, function, and OHRQoL following disc repositioning. According to a recent network meta-analysis, when combined with intra-articular hyaluronic acid or platelet-rich plasma, arthrocentesis provides significant short-term pain relief. However, these effects diminished within 6–12 months with only moderate gains in function and OHRQoL [30].

Similarly, a randomized trial of mandibular exercise therapy reported only modest improvements in VAS-P (2–3 points) and OHIP-14 (3–5 points) at 6 months [31].

It is important to note that the minimally invasive trials included patients with early or less refractory DDwoR, whereas the present cohort consisted exclusively of refractory bilateral DDwoR cases, indicating that mini-anchor fixation was associated with substantial clinical improvement in this refractory cohort. Nevertheless, these comparisons are indirect and should be interpreted with caution. Furthermore, clinicians and patients should maintain realistic expectations; it is unrealistic to expect that any TMJ surgery will completely eliminate all signs and symptoms or fully restore normal TMJ function [10]. However, the observed improvements in function and OHRQoL are encouraging.

At 12 months, outcomes were both substantial and clinically significant: OHIP-14 decreased by 76%, VAS-P by 91%, and DI by 66%. Improvements in OHIP-14 and VAS-P exceeded the prespecified MCIDs (OHIP-14 ≥ 4.0 points; VAS-*P* ≥ 2.0/10), while DI showed consistent functional improvement over time. Patients in both age groups moved from severe to mild dysfunction. These subjective improvements were supported by objective data, as significant postoperative improvements in mandibular mobility—including maximum opening, laterality, and protrusion (*p* < 0.001)—were consistent with functional improvement following TMJ disc repositioning in DDwoR.

The transient decrease at T1 aligns with early postoperative edema and protective limitation, with recovery observed at T2. Incorporating both subjective and objective parameters may provide a more comprehensive assessment of treatment effectiveness. While surgical expertise remains a key factor in success, measuring OHRQoL is essential for understanding patient-centered outcomes [32]. This approach not only facilitates comparisons with different procedures but also improves the way outcomes are measured in clinical trials, supporting evidence-based clinical decision-making [33].

This study supports the view that TMJ disc repositioning can be considered successful when it is associated with improved mandibular mobility, less pain, and better OHRQoL. While some symptom improvement may be part of the natural course of internal derangement, these results reinforce existing evidence supporting disc repositioning in carefully selected cases. Therefore, for individuals who do not improve after a well-conducted course of conservative therapy, the magnitude of the functional and OHRQoL improvements observed suggests that disc repositioning may be a viable therapeutic option.

While our findings emphasize the benefits of disc repositioning, recent literature proposes that synovitis, rather than disc position [21], may be the primary driver of TMD symptoms, and the observed improvements may be linked to indirect inflammation reduction [34]. The lack of a non-surgical control group limits causal attribution, and future randomized trials with inflammatory markers are needed to clarify these effects.

### Study limitations

Although the OHIP-14 and VAS-P are validated tools, their subjective nature may introduce recall and interpretation bias. Therefore, the DI was employed to provide objective clinical support. Because of the surgical nature of the intervention, blinding of patients and outcome assessors was not feasible. The relatively short follow-up and small sample size (*n* = 34) were sufficient for statistical significance, but limited generalizability and subgroup analysis. The main limitation is the lack of a control group, since the cohort consisted exclusively of patients with refractory bilateral DDwoR after prolonged conservative therapy. Therefore, the present findings should be interpreted as longitudinal clinical outcomes observed after intervention rather than as causal estimates of treatment effect. Part of the observed improvement may reflect natural history, regression to the mean, contextual or placebo effects, as well as the contribution of postoperative physiotherapy and behavioural adaptation. Natural history studies have reported spontaneous symptom resolution in 40–60% of untreated DDwoR patients over 12–30 months, with lower rates of spontaneous improvement in older patients and in those with prior treatment failure [35, 36]. Given that the present cohort consisted exclusively of patients who had already failed ≥ 6 months of structured conservative therapy, the expected contribution of spontaneous remission is plausibly below the lower bound of this range. The magnitude of improvement observed here — 91% reduction in VAS-P and 76% in OHIP-14 — appears greater than these estimates, although causal attribution is not possible without a control group. A randomized design was not pursued because withholding surgery from patients with documented conservative treatment failure and MRI-confirmed derangement would raise ethical concerns in this refractory surgical cohort. An additional limitation is the absence of routine postoperative MRI confirmation of disc position at follow-up; therefore, the present study cannot determine whether the observed clinical improvement was directly related to persistent anatomical disc repositioning over time. A prespecified non-parametric sensitivity analysis for the ordinal DI category score yielded results consistent with the primary analysis. Further stratification by adherence was not applicable, as all participants met the same ≥ 6-month conservative therapy threshold; stratification by symptom duration was precluded by incomplete prospective documentation of this variable.

## Conclusion

TMJ disc repositioning with mini-anchor fixation was associated with significant improvements in OHRQoL, pain, clinical dysfunction, and mandibular mobility over 12 months in women with refractory bilateral DDwoR. Benefits were evident within 30 days and remained sustained at final follow-up. Because of the absence of a concurrent control group, these findings should be interpreted as postoperative clinical associations rather than causal treatment effects. Further controlled studies are needed to validate these findings.

## Data Availability

The datasets used and/or analyzed during the current study are available from the corresponding author on reasonable request.

## Declarations

### Strobe statement

Checklist of items that should be included in reports of observational studies.
